# Association between children living with obesity and Mental Health problems: a data analysis of the Welsh Health Survey, UK

**DOI:** 10.1186/s12889-023-15293-8

**Published:** 2023-02-23

**Authors:** Claire Beynon

**Affiliations:** Public Health Wales, Capital Quarter 2, Tyndall Street, CF10 4BZ Cardiff, UK

**Keywords:** Obesity, Epidemiology, Public Health, Pediatrics, Adolescent

## Abstract

**Background:**

Obesity and mental health problems in children are both significant and growing public health issues. There is mixed evidence on the relationship between obesity and mental health in children. This study examines the association between obesity and mental health problems in a nationally representative sample of children using the Welsh Health Survey for Children (n = 11,279 aged 4–15 years).

**Methods:**

The Chi-square test assessed the difference in the proportion of children reporting abnormal mental health scores (strengths and difficulties score ≥ 20) in children living with obesity (≥ 95 centile for age and sex). Then, a multivarible logistic regression was used to assess any association after accounting for confounding variables.

**Results:**

There were 1,582 children living with obesity in the study (19.6%). The Chi-square test indicated a significant difference in the proportion of children with abnormal mental health scores in children living with obesity (p = 0.001). This study found a very small but significant positive association between mental health and childhood obesity after accounting for confounding variables, Odds Ratio 1.02 (95%CI: 1.01 to 1.02, p = 0.001). However, socio-economic status was more of a driver.

**Conclusion:**

The findings of this study show a very small but significant association between childhood obesity and mental health problems. The multivariable logistic regression indicates that the focus must remain on reducing health inequalities as this is a more important driver of child health and well-being. However, as a precautionary measure it may be worth considering if children living with obesity who present for weight-management services may benefit from a review of their mental health status to identify if further support is needed, if capacity allows, and this can be done in a supportive way.

## Introduction

This study fulfils a need identified in a recent report from the United Kingdom (UK) Chief Medical Officers [1] which suggested more research was needed to investigate the relationship between childhood obesity and mental health. It is essential to understand any association to inform clinical evaluation and treatment plans and optimise pathways for both disorders. Both obesity and common mental health disorders account for a significant proportion of the global burden of disease [2].

Childhood obesity has increased worldwide over the last 30 years [3]. Childhood obesity in Wales, UK continues to rise and has now reached 12% at age 5 [4]. Obesity has a considerable cost to the health service, economy and society [5]. Childhood obesity often persists into adulthood where most negative health impacts are observed [6]. A meta-analysis by Simmonds et al. (2016) found that children living with obesity were five times more likely to experience obesity in adulthood [7]. Childhood obesity can also result in substantial threats to health and well-being throughout the life course [8]. Children living with obesity are more likely to develop non-communicable diseases like diabetes and cardiovascular diseases at a younger age and childhood obesity can affect children’s physical health, social, and emotional well-being, and self-esteem [9, 10]. Obesity is also associated with poor academic performance and a lower quality of life [11]. About one third of girls who are overweight and a quarter of boys who are overweight report being teased by peers at school [12]. Hence, children living with obesity often face stigmatisation and discrimination in many areas of their lives [13]. Obesity has been shown to reduce a child’s opportunities to engage in activities and learning at home and in school, especially social participation and play [14]. The COVID-19 pandemic has increased levels of obesity in children [15]. In addition, in developed countries, obesity is regularly reported to be highest in children from the lowest socioeconomic strata, therefore any analysis should include deprivation as a confounding variable [16].

In the UK, one in ten children have a clinically diagnosed mental health disorder and/or emotional and behaviour problems [17]. In both the short and long term, children with poor mental health have an increased likelihood of poor social and economic outcomes [18]. The number of children seeking help for mental health problems has increased in Wales [19]. Furthermore, poor mental health in childhood is associated with an increased risk of self-harm and suicide [20]. Half of lifetime mental health problems start by the age of 14, it is therefore important to explore the relationship between obesity and mental health in the paediatric population [21]. The World Health Organization describe a 10–25 year life expectancy reduction in patients with severe mental disorders [22]. The rate of increasing requests for the treatment of mental health problems in children creates a considerable challenge for health services in children [23]. The demand for mental health services and difficulties in providing COVID-19 secure services exacerbated this [24]. In 2009, the cost of mental health problems in Wales was estimated at £7.2 billion/year, this together with the human cost, make it an important public health issue [25]. In developed countries, poor mental health is found at higher rates in children of the lowest socioeconomic status, it is therefore important to include deprivation as a confounding variable in any analysis [26].

Whether there is a relationship between obesity and mental health is still uncertain with conflicting evidence [27, 13]. The original contribution of this paper is that it is uses a large, generalisable, population-based study to examine the association between obesity and mental health in children. Existing studies often focus on small, clinical populations [28–31]. Indicating a need to further investigate the relationship between obesity and mental health at the population level [32]. The relationship specifically between one aspect of mental health, that of depression, and childhood obesity has been established in a recent meta-analysis [33]. However, there is limited research on the relationship between obesity and overall mental health: Russell-Mayhew et al. 2012 concluded that the direction of the relationship between obesity and mental health remains unclear [34]. The aim of this study was therefore to identify and quantify any cross-sectional associations between obesity in children and abnormal mental health scores (using the Strengths and Difficulties Questionnaire) in a large national dataset, after accounting for deprivation and other confounding variables. In this study deprivation was measured using the Welsh Index of Multiple Deprivation which is a composite measure that includes levels of crime, employment, income, education, housing, access to services, the environment and overall health in the local area. The Children’s Welsh Health Survey was used as it has not previously been utilised for this purpose [35].

## Methods

The sample frame for the Children’s Welsh Health Survey is all residential homes in Wales, selected from an up-to-date list of addresses in the Post Office™ Postcode Address File [36].

### Ethics statement

Data collection was approved by the Research Ethics Committee of the National Centre for Social Research, UK. The research design and ethical considerations are thorough and conform to the required procedures. All methods were carried out in accordance with relevant guidelines and regulations. All participants provided their informed consent on research participation before entering the survey. Informed consent was obtained from the parent(s)/guardian(s) of all under-16’s. The participants are assured that their personal information will remain confidential and will not be disclosed. The present study was conducted in accordance with the principles of the 2013 Declaration of Helsinki.

### Subjects and design

The Children’s Welsh Health Survey collected data from 3,000 children each year between 2008 and 2012 using a stratified sampling technique to produce a nationally representative sample. The Children’s Welsh Health Survey was conducted in the child’s home, however, occasionally if the family was unable to fully complete the survey with the trained interviewer, the survey was initiated in the home where children had height and mass measured and then the family return the survey by post, at their convenience. Parents of children aged 4–12 answered the survey on behalf of their child, whilst children aged 13–15 completed the survey independently. The response rate was 79%.^35^ The survey questions were subject to cognitive testing to ensure that they are consistently understood as intended, and respondents could recall the information needed to answer them [35]. To avoid seasonal variation, data were collected throughout the year.

### Mental Health Questionnaire

The Strengths and Difficulties Questionnaire (SDQ) is a validated screening tool used to assess both behavioral and mental health difficulties in children [37]. The SDQ contains 25 statements, five questions on each of the following five topic areas: conduct problems, hyperactivity, emotional symptoms, peer problems and pro-social behaviours. The respondent considers each of the 25 statements, e.g. “Considerate of other’s feelings”, and three possible answers: Not True, Somewhat True or Certainly True. The SDQ has high test-retest reliability and good validity [38]. The SDQ provides an overall assessment of mental health using the sum of the difficulties scores (conduct problems, emotional symptoms, hyperactivity, peer problems). A higher score indicates more mental health difficulties, a score equal or greater than 20 indicates mental health difficulties. The SDQ has been validated against semi-structured diagnostic interviews in terms of the ability to detect clinically significant behavioral or emotional disturbance in the community setting [39]. Full details of the SDQ tool [40] and norms are available at http://sdqinfo.org/.

### Obesity measure

Trained interviewers recorded the height of each child to the nearest millimeter (Marsden HM-250P Leicester Portable Stadiometer) and the mass of each child (without shoes or outdoor clothing) in kilograms to one decimal place (Tanita THD-305 scales). Mass and height were used to generate a Body Mass Index (BMI) score for each child. Obesity was classified using sex and age with BMI equal to or greater than the 95th centile. The 95th centile is used as the definition for childhood obesity in the UK [41] the International Obesity Task Force [42].

The data set was cleansed: all subjects with a BMI of under 13 kg/m^2^ were removed (n = 45); all children with a BMI over 35 kg/m^2^ (n = 56) were reviewed with a paediatrician to assess the raw height and weight data with reference to their age and sex and five high BMI outliers where data was not plausible were removed. All remaining data were used in the descriptive and analytical statistics. Comparison was made between children living with obesity and all other children (normal weight and overweight children): this is in line with guidance from previous authors [43].

### Measure of deprivation

The relationship between obesity and deprivation is well documented [44]. It was therefore important to account for deprivation at the analysis stage, this was managed using the Welsh Index of Multiple Deprivation (WIMD). The WIMD is the approved measure of deprivation in small areas in Wales [45] WIMD is not a direct measure of deprivation, but the deprivation rank is determined by the circumstances and lifestyles of the people living in that area. The WIMD category was classified according to the postcode of the main dwelling of each child.

### Statistical analyses

The Chi-square test was used to establish if there was any difference in the proportions of children reporting clinically significant mental health scores (≥ 20) between children living with obesity and children without obesity. The results are reported with levels of significance.

Logistic regression was utilised to explore the relationship between obesity and mental health as this methodology can account for confounding variables. Complete case analysis was used: there was some missing data (BMI available for n = 8,078). However, the analysis of those with missing BMI data in comparison to those with complete data showed no significant differences in a range of other variables including SDQ scores. Univariate logistic regression was undertaken using obesity and the following variables: mental health, daily vegetable consumption, WIMD quintile [45] and physical activity as these are all highlighted as potential confounding variables in the literature [46, 26, 47]. The univariate and multivariable logistic regression were undertaken by age groupings for self-report participants (13–15 years) and parental report participants (4–12 years) due to the different data collection methodology for these groups. The Odds Ratios and 95% Confidence Intervals (CI) were calculated and reported. All statistical analysis was undertaken using STATA/IC 14.2.

## Results

### Characteristics of the study sample

The distribution of the sample by age, sex, WIMD quintile, BMI category and lifestyle variables is provided in Table 1. The sample used a stratified sample with approximately 1,000 children in each age group from 4 to 15 years, mean age 9.6 years, Standard Deviation ± 3.5 years. The percentage split of girls (48.5%) and boys (51.5%) is similar to the childhood population for Wales [48].

**Table 1:** Summary Statistics of the Stratified Sample

Variable	Data recorded	Number (percentage)
**Sex recorded** Male Female	11,279	5,813 (51.5) 5,466 (48.5)
Age Age 4 Age 5 Age 6 Age 7 Age 8 Age 9 Age 10 Age 11 Age 12 Age 13 Age 14 Age 15	11,279	949 (8.4) 900 (8.0) 908 (8.1) 844 (7.5) 931 (8.3) 889 (7.9) 905 (8.0) 943 (8.4) 958 (8.5) 987 (8.8) 1,023 (9.1) 1,042 (9.2)
**WIMD (calculated from postcode**) Quintile 1 Quintile 2 Quintile 3 Quintile 4 Quintile 5	11,279	2,193 (19.4) 2,262 (20.1) 2,344 (20.8) 2,187 (19.4) 2,293 (20.3)
**BMI recorded** Normal Weight Overweight Obese	8,078	5,186 (64.2) 1,310 (16.2) 1,582 (19.6)
**Physical activity recorded** Active for ≥ 1 h, Daily	11,014	5,822 (52.4)
**Diet** Daily Vegetable Consumption	11,126	5,747 (51.0)

### Descriptive statistics for obesity

A total of 1,582 (19.6%) of children in this sample, were children living with obesity. There was a small difference in prevalence of obesity by sex in the sample, with boys having higher levels n = 897 (21.1%) than girls n = 685 (17.9%). The percentages of normal weight children were similar by sex with 45.9% boys compared to 46.1% girls (Table 1). The univariate logistic regression of weight classification by gender showed no statistically significant difference and therefore sub-group analysis by gender was not undertaken.

### Descriptive statistics for strength and Difficulty Questionnaire

The differences in the SDQ by obesity classification are described in Table 2. This shows the mean score for the children living with obesity was almost one point higher at 8.5, compared to other children. When considering abnormal SDQ scores (≥ 20), the difference between the children living with obesity and children without obesity was 2.1% (Table 3). This was a statistically significant proportion in the Chi-square test (p = 0.001).

**Table 2:** Mean and Standard Deviation of the Strengths and Difficulty Scores (SDQ) for Children aged 4–15 by Body Mass Index Category

Body Mass Index (BMI) classification	SDQ mean	SDQ SD	Frequency
**Obese**	8.5	6.4	1,582
**Normal weight**	7.6	5.9	5,186
**Overweight**	7.6	5.9	1,310
**No BMI data**	8.0	6.4	3,201
**TOTAL**	7.8	6.1	11,279

Cohen’s d = 0.15

**Table 3:** Strengths and Difficulty Scores (SDQ) Under 20 and Greater than 20 by Body Mass Index Category

	Body Mass Index (BMI) classification	
	Non-obese (percentage)	Obese (percentage)
**SDQ score under 20**	6,151 (95.7)	1,468 (93.6)
**SDQ score 20 or more**	276 (4.3)	100 (6.4)
**TOTAL**	6,429 (100)	1,568 (100)
Chi Square Test used all available data = 0.001		

### Confounding variables

Table 1 shows children in the sample who reported being physically active daily was n = 5,822 (52.4%). Obesity in those who were active was lower at 17.7% compared to 21.8% of children who were not. The univariate logistic regression indicated this was statistically significant (Table 4).

Table 4 details the univariate Odds Ratios and 95%CI. The variables that were statistically significant in the univariate logistic regression and were therefore included in the multi-variable logistic regression were: WIMD; being active and daily vegetable consumption.

There was an increasing rate of obesity as social deprivation increased, from n = 243 (15.7%) in the least deprived group to n = 347 (22.3%) in the high deprivation group. Deprivation was statistically significant in the univariate and multivariable logistic regression (Tables 4 and 5).

**Table 4:** Univariate Logistic Regression of Childhood Obesity with Variables

n = 10,088	Odds Ratio	(95% CI)	p-value
Mental Health (SDQ difficulties score)	1.02	1.01–1.02	0.001*
Sex	0.99	0.93–1.08	0.94
WIMD Quintile 1	Reference		
WIMD Quintile 2	1.14	1.01–1.29	0.03*
WIMD Quintile 3	1.28	1.13–1.44	0.001*
WIMD Quintile 4	1.30	1.15–1.47	0.001*
WIMD Quintile 5	1.51	1.34–1.70	0.001*
Daily Vegetable Consumption	0.94	0.91–0.96	0.001*
Physical Activity Daily	0.95	0.92–0.97	0.001*

*denotes statistically significant at p < 0.05 level

**Table 5:** Multivariable Logistic Regression of Childhood Obesity with Mental Health Adjusted for Deprivation and Lifestyle Factors for Children 4–15 years

n = 10,934	Odds Ratio	(95% CI)	p-value
Mental Health (SDQ difficulties score)	1.02	1.01–1.02	0.001*
WIMD Quintile 1	Reference		
WIMD Quintile 2	1.11	0.98–1.26	0.098
WIMD Quintile 3	1.24	1.10–1.40	0.001*
WIMD Quintile 4	1.21	1.07–1.37	0.001*
WIMD Quintile 5	1.39	1.23–1.58	0.001*
Daily Vegetable Consumption	0.86	0.80–0.93	0.001*
Physical Activity Daily	0.83	0.77–0.90	0.001*

*denotes statistically significant at p < 0.05 level

### Multivariable logistic regression

The multivariable logistic regression showed obesity was associated with poor mental health even after accounting for deprivation and lifestyle with a very small but statistically significant Odds Ratio, OR 1.02 (95% CI: 1.01 to 1.02, p > 0.001) see Table 5 for full details. When analysed by age, the self-report group (13–15 years) results were non-significant OR = 1.01 (95%CI 0.99–1.02, p = 0.218) whereas the results for the younger children (4–12 years) was similar to the overall findings with OR = 1.01 (95%CI 1.01–1.02, p = 0.003).

## Discussion

The main findings of this study are twofold. Firstly, the results show a statistically significant difference between the proportion of children who record clinically significant SDQ scores (≥ 20) in children living with obesity when compared to other children. Secondly, there is a very small but statistically significant association between children living with obesity and mental health after accounting for confounding variables in this nationally representative dataset. The direction of these results indicates a positive association between obesity and poorer mental health scores (i.e. higher SDQ scores in children living with obesity).

The percentage of children living with obesity and severe obesity is rising [54] and the global pandemic has exacerbated this trend [16]. The mean and standard deviation of the SDQ scores were similar to norms provided for UK children [49]. The mean SDQ score for the children living with obesity was almost one point higher. A score one point higher on the SDQ scale is deemed a clinically relevant difference, [34] (noting that this difference was observed prior to adjusting for confounding variables). The difference in SDQ scores between the children living with obesity and other children in this study is therefore both clinically and statistically significant. This is comparable to the study by Griffiths et al. 2011 who identified similar differences in SDQ scores to those found in this study in children at age five [50].

The public health implication could use the precautionary principle, [51] i.e. to exercise precaution while dealing with uncertainty [52]. Whilst there is uncertainty about the direction of the relationship between obesity and mental health, public health action may include consideration of whether an assessment of mental health could be beneficial when obesity is identified in a child by a healthcare practitioner. This supports the latest UK public health advice on the management of obesity in children which recommends, “if concerns about their mental wellbeing are identified refer the child or young person to their GP for assessment and treatment and, if appropriate, for onward referral to child and adolescent mental health services.” [53] Childhood obesity is a complex and persistent public health issue [54]. The Welsh Government has responded to this by publishing its first Obesity Strategy for Wales, which outlines the strategies to be employed in the next decade to tackle obesity, including identification and treatment of children living with obesity [55].

A total of 1,582 (19.6%) of children were living with obesity, with obesity rates increasing with age in line with national trends [56]. This is similar to the findings Millennium Cohort Study where 20% of children were living with obesity by the age of eleven [57]. In the current study we compared children living with obesity to all other children (including all children classified as either normal weight or overweight children), as recommended by previous authors who suggest that the combination of obesity and overweight may weaken any conclusions [43]. This was appropriate as the study set out to explore the association between children living with obesity and mental health problems. If children with overweight had been grouped with those categorised as obese it would have been more difficult to see the specific relationship between obesity and mental health. With overweight and obesity increasing in Wales [54] not being a healthy weight is becoming normalised in children, making it important to differentiate obesity from overweight. From Table 1 we can see that when combined 35.8% of children were classified as either overweight or obese. The observation that there is a greater risk of obesity in children from more deprived groups (OR ranging from 1.11 to 1.40) is in line with other UK literature [1, 39]. This adds credence to the results of the study and indicates that policies that reduce inequalities remain the most important area of focus for tackling obesity in children.

The number of population-based studies that review the relationship between obesity and mental health are very limited. The literature in this field shows mixed results [34, 31, 58]. One large cross-sectional study in America (n = 4,743), [59] found a statistically significant relationship between BMI and physical health but not psychosocial outcomes except in the youngest group (12–14 years) this is in line with the findings of the current study. A cross-sectional study [60] in France (n = 1,026 6–11 year olds) reported a statistically significant difference in abnormal SDQ scores for children living with obesity, but the authors did not adjust for confounding variables, making comparison with the current study difficult. Another study [61] in Norway combined data from two cross-sectional studies undertaken in 2002 and 2017 (n = 3,188) and found a small but significant relationship, but when this was adjusted for confounding variables the results were non-significant. The relationship between obesity and mental health scores found in the current study is similar in scale and direction to that found in other studies, with the exception that in the current study the relationship is maintained after accounting for confounding variables. This could be due to the scale of our study or different cultures in different countries. Even across the four countries in the UK we can see that childhood obesity varies, with Wales consistently having the highest rates of obesity in children in the four countries over the last few decades [58].

From the existing literature examining the reasons why there might be a relationship between obesity and poor mental health there are numerous theories but most indicate that they appear to exist in a causal loop, with each exerting cause and effect influences on each other [62, 63]. Sagar and Gupta (2018) describe children living with obesity as experiencing more discrimination, social isolation and bullying which has an impact on mental well-being [62]. Whilst Smith et al. (2020) describe that children with poor mental well-being are often characterised as withdrawing from physical activity and binge eating which in turn increases weight in children, and state that the direction of the relationship between mental health and obesity is uncertain [63]. Any planned interventions should therefore be mindful of this bidirectional relationship.

The results in this study differed by age group. For the (4–12 years) the results were similar to the results for the whole group, this is as expected as it made up the majority of the participants 72.9% (n = 8,227). However, the non-significant result for the older children (13–15 years) who self-reported scores indicated that obesity and total SDQ scores were not associated in this group, perhaps indicating that this relationship may become less important with age. It is unlikely that this is due to the different data collection methods (i.e. parental-report for children up to 12 and self-report for those 13–15 as both methods have been validated for these age groups, and the studies that have assessed the correlation of youth and parent scoring have shown favourable parent-youth agreement [37–39, 64].

The main strength of this study is the large data set (n = 11,279) which confers an advantage over previous studies that report outcome results based upon small samples. The use of stratified random sampling aids generalisability to the wider population. Once enough primary studies are undertaken a meta-analysis should be conducted to settle the controversies arising from the conflicting results presented in the literature.

Further strengths of the study were that the height and mass of each child was measured by a trained interviewer rather than relying on self-report which improves the accuracy of the BMI data [65]. The study also used multivariable logistic regression to account for confounding variables. The main limitations of this study are the cross-sectional design, and the timeframe of the data collection. Cross-sectional data can identify associations, but it is not possible to ascribe any causal relationships from cross-sectional data. Whilst the data was collected between 2008 and 2012 the results are still important to report and relevant as both obesity and mental health issues have deteriorated since then in the UK and beyond. In future, longitudinal data sets should be used to explore this relationship. However, longitudinal studies often have fewer participants and are expensive to run. There was no more up to date data for Wales that had been collected with all of the variables needed to undertake this analysis. A recommendation has therefore been made to the Welsh Government to add to BMI and SDQ to surveillance systems. A further limitation of the study is that the age range of the children used two different data collection techniques, parental report for children 5–12 years and self-report for children aged 13–15 years. This follows the guidelines for use of the questionnaire. To mitigate this, statistical analyses were run for children 5–12 and 13–15 separately. With increasing rates of obesity and mental health problems in childhood globally, an understanding of the relationship between obesity and mental health assumes even greater importance.

This study shows a very small but significant association between obesity and mental health problems. Whilst the association is small, it is both clinically and statistically significant, and as a precautionary measure, consideration could be given to a psychological element to obesity services that are being planned for children using the precautionary principle. However, the main focus must remain on reducing inequalities in society.

## Conclusion

This study explored the association between childhood obesity and mental health problems in children that exists above and beyond deprivation status. Whilst the relationship identified between obesity and mental health is very small it may be prudent to consider the review of mental health status in children entering weight management programmes as a precautionary measure to assess if further complementary mental health support is needed. However, this should only be attempted if it does not cause harm, i.e. in a non-stigmatising way. In addition, this study has demonstrated that there are statistically significant poorer health outcomes in relation to both obesity and mental health for children in lower socio-economic groups, demonstrating that policies that reduce the number of children living in poverty are crucial. Findings from this study will be of interest to public health professionals, policy makers and providers of obesity and mental health services for children.

## Data Availability

The data that support the findings of this study are available from the Welsh Government but restrictions apply to the availability of these data, which were used under a formal data access agreement for the current study, and so are not publicly available. Data are however available from the authors upon reasonable request and with permission of the Welsh Government.
